# TMEM147 Correlates with Immune Infiltration and Serve as a Potential Prognostic Biomarker in Hepatocellular Carcinoma

**DOI:** 10.1155/2023/4413049

**Published:** 2023-06-03

**Authors:** Sheng Cheng, Jutang Li, Ming Xu, Qun Bao, Jiaoxiang Wu, Peng Sun, Bo Han

**Affiliations:** ^1^Department of General Surgery, Tongren Hospital, Shanghai Jiao Tong University School of Medicine, Shanghai 200336, China; ^2^Key Laboratory for Translational Research and Innovative Therapeutics of Gastrointestinal Oncology, Hongqiao International Institute of Medicine, Tongren Hospital, Shanghai Jiao Tong University School of Medicine, Shanghai 200336, China; ^3^Department of Clinical Laboratory, Tongren Hospital, Shanghai Jiao Tong University School of Medicine, Shanghai 200336, China

## Abstract

**Background:**

Hepatocellular carcinoma (HCC) is one of the most prevalent malignancies and is associated with high mortality. Transmembrane protein 147 (TMEM147) is a seven-transmembrane protein that may mediate immune regulation. However, the relevance of TMEM147 to immune regulation in HCC and the prognosis of HCC patients are unclear.

**Methods:**

We analyzed TMEM147 expression in HCC by using the Wilcoxon rank-sum test. Real time quantitative PCR (RT-qPCR) and Western blot analysis of tumor tissues and cell lines were used to verify TMEM147 expression in HCC. The influence of TMEM147 on HCC prognosis was assessed using Kaplan–Meier analysis, Cox regression analysis, and a prognostic nomogram. The functions of the TMEM147-related differentially expressed genes (DEGs) were identified by Gene Ontology (GO)/Kyoto Encyclopedia of Genes and Genomes (KEGG) enrichment analyses and gene set enrichment analysis (GSEA). In addition, we examined the associations between TMEM147 expression and immune infiltration using single-sample gene set enrichment analysis (ssGSEA) and immunofluorescence staining of HCC tissues.

**Results:**

Our results showed that the expression of TMEM147 was significantly higher in human HCC tissues than in adjacent normal liver tissues, with similar findings in human HCC cell lines. High TMEM147 expression was correlated with T stage, pathological stage, histological grade, race, alpha-fetoprotein level, and vascular invasion in HCC. Moreover, we revealed that high TMEM147 expression was associated with shorter survival times and that TMEM147 could be a risk factor for overall survival, along with T stage, M stage, pathological stage, and tumor status. Mechanistic studies revealed that high TMEM147 expression was linked to the B lymphocyte, antigen response, IL6 signaling pathway, cell cycle, Kirsten rat sarcoma viral oncogene homolog (KRAS) signaling pathway, and myelocytomatosis oncogene (MYC) targets. Correspondingly, TMEM147 expression was positively associated with the infiltration of immune cells, including Th2 cells, follicular helper T cells, macrophages, and NK CD56 bright cells in HCC.

**Conclusions:**

TMEM147 might be a biomarker for poor prognosis and is related to immune cell infiltration in HCC.

## 1. Introduction

Hepatocellular carcinoma (HCC) is the fourth leading cause of cancer death worldwide, with 700,000 related annual deaths in recent years, half of which occurred in China [1, 2]. Risk factors for HCC development include viral infections [3], alcohol abuse, and genetic factors [4, 5]. With improvements in medical technology, the global morbidity and mortality of HCC have recently decreased [6], but the recurrence rate (>70%) and relapse-related mortality remain high [7]. Additionally, the molecular mechanisms of HCC have not yet been fully elucidated. Thus, there is an urgent need to understand HCC progression, which may lead to discovering a new therapeutic agent to improve patient survival.

Transmembrane protein 147 (TMEM147) is a 22 kDa protein encoded by a gene located on chromosome 19q13.12 [8]. As an endoplasmic reticulum (ER) resident protein with seven transmembrane regions, TMEM147 is highly conserved in mammals, but its physiological role is not fully understood. Previous work has shown that TMEM147 is a core component of the nicalin–NOMO protein complex and is essential for embryonic Nodal signaling during embryonic development in vertebrates [9]. Recent research has demonstrated that TMEM147 is a component of a multipass translocon that supports the biogenesis of membrane proteins on the ER-bound ribosomes [10]. Additionally, TMEM147 can mediate immune regulation by binding to galectins of *Haemonchus contortus* in goats [11]. Downregulation of TMEM147 suppresses cytokine-induced rheumatoid arthritis (RA) by inhibiting the activation of nuclear factor kappa B signaling [12]. Emerging evidence reveals the vital roles of TMEM147 in tumor occurrence and progression. A recent study reported that high expression of TMEM147 may contribute to the development of colon cancer [13]. Similarly, in osteosarcoma, the prognostic marker TMEM147 has been linked to immune cell infiltration and the immunological microenvironment [14]. Another functional illustration revealed that TMEM147 adversely controlled calcium mobilization caused by the cholinergic receptor muscarinic 3 (CHRM3) and interfered with its trafficking to the cell membrane in colon cancer [15]. It's interesting to note that CHRM3 is aberrantly expressed in several malignancies, including HCC, and is regarded to be a prognostic factor for these diseases [16–18]. Based on these findings, TMEM147 may play a key role in immune regulation and contribute to tumor progression. However, the functional role of TMEM147 in HCC has not been delineated. Furthermore, whether and how TMEM147 mediates immune regulation in HCC remains unknown.

In the present study, we analyzed RNA sequencing data from The Cancer Genome Atlas (TCGA) database to investigate the expression of TMEM147 and its prognostic value in HCC. Gene Ontology (GO) term enrichment analysis, Kyoto Encyclopedia of Genes and Genomes (KEGG) pathway enrichment analysis, and gene set enrichment analysis (GSEA) of the HCC dataset from TCGA were applied to further identify the role of TMEM147 in the emergence of HCC. Finally, the associations between TMEM147 expression in HCC and tumor immune cell infiltration were investigated to evaluate the underlying mechanisms of TMEM147.

## 2. Materials and Methods

### 2.1. Data Acquisition

RNA-seq transcriptome data for HCC samples (374 tumor tissues and 50 paracancerous tissues) with the clinical information of the corresponding patients were obtained from the TCGA database (https://portal.gdc.cancer.gov/). The HTSeq-FPKM level 3 data were converted to TPM (transcripts per million reads) values for further analysis. Gene expression profile data of HCC were obtained from the Gene Expression Omnibus (GEO) database (https://www.ncbi.nlm.nih.gov/geo/) for the GSE101685 (8 normal tissue and 24 HCC samples; Platform: GPL887); GSE62232 (10 normal liver samples and 81 HCC tissues; Platform: GPL570); and GSE60502 (18 pairs of HCC and adjacent nontumor liver tissues; Platform: GPL96) datasets. Data analysis was conducted using R software, and our study fully complied with the guidelines published by TCGA and GEO.

### 2.2. Clinical Samples and Cell Cultures

HCC tissues and corresponding adjacent normal tissues from 16 patients were provided by Tong Ren Hospital, Shanghai Jiaotong University School of Medicine, and the tissues were collected between 2020 and 2022 and diagnosed histopathologically. The tissue samples were stored in liquid nitrogen for long-term preservation. Before sample collection, written informed consent was provided by all participating patients. The study was approved by the ethics board of Shanghai Tongren Hospital (No. 2020-035-01). Five human HCC cell lines (SK-HEP-1, MHCC-97H, SMMC-7721, HepG2, and Hep3B) and the human normal liver epithelial cell line L02 were obtained from the Chinese Academy of Sciences. All cells were cultured in Dulbecco's modified Eagle's medium (DMEM; Cat. C11995500BT, Gibco) supplemented with 10% fetal bovine serum (FBS; Cat. S711-001S, NSERA) and 1% penicillin/streptomycin (Cat. 10378016, Gibco) at 37°C in a 5% CO_2_ incubator.

### 2.3. RNA Isolation and Real-Time qPCR

Total RNA was extracted from tissues and cells with RNAiso Plus (Cat. 9109, TaKaRa, Japan) based on the manufacturer's protocol. ReverTra Ace® qPCR RT Master Mix with gDNA Remover (Cat. FSQ-301, TOYOBO, Japan) was used to synthesize cDNA. Real time quantitative PCR (RT-qPCR) was performed with ChamQ Universal SYBR qPCR Master Mix (Cat. Q711-02, Vazyme, China). The primer sequences used in this study for amplifying TMEM147 and *β*-actin were as follows: TMEM147 (forward, 5′-ACACGCTATGATCTGTACCACA-3′, reverse, 5′-CAGAGGTGGACGAAGGTCTC-3′) and *β*-actin (forward, 5′-CATGTACGTT GCTATCCAGGC-3′, reverse, 5′-CTCCTTAATGTCACGCACGAT-3').

### 2.4. Analysis of Differentially Expressed Genes in Patients with HCC

A total of 374 HCC patients were categorized into the high and low TMEM147 mRNA expression (cutoff value of 50%) groups. Differentially expressed genes (DEGs) between these two groups were identified using the Wilcoxon rank-sum test in the R package “DESeq2” [19]. The threshold criteria of |log2Fold Change| > 1.5 and *p* < 0.05 were used to identify DEGs. A heatmap was generated to show the top 20 DEGs.

### 2.5. Functional Enrichment Analyses

The DEGs were subjected to functional enrichment analyses. The GO term enrichment analysis for the biological process (BP), molecular function (MF), and cellular component (CC) categories, as well as KEGG pathway enrichment analysis, was performed by using the R package “cluster Profiler”, and the results were visualized with the “ggplot2” package in R [20, 21].

### 2.6. Gene Set Enrichment Analysis

Novel BPs and pathways differing between the high and low TMEM147 expression groups were identified using GSEA. GSEA was conducted using the R package clusterProfiler (3.14.3) [22]. Each analysis was repeated 5,000 times. In this study, *p* < 0.05, FDR < 0.25 and absolute normalized enrichment score (|NES|) > 1 were used as the criteria for statistical significance.

### 2.7. Associations between TMEM147 Expression and Immune Cell Infiltration in HCC

To analyze the associations of TMEM147 expression with immune infiltration, single-sample gene set enrichment analysis (ssGSEA) was performed with the “GSVA” R package [23]. As described previously, we evaluated the relative infiltration levels of 24 immune cell types [24]. The relative enrichment score of each gene expression profile was calculated for each tumor sample. For analysis of associations between immune cell infiltration and TMEM147 expression, the Wilcoxon rank-sum test and Spearman correlation analysis were used.

### 2.8. Western Blot Analysis

Proteins were extracted from tissues using lysis buffer (50 mM Tris–HCl, 150 mM NaCl, 1% NP-40, 0.5% sodium deoxycholate, and 0.1% sodium dodecyl sulfate (SDS); pH 7.5) in the presence of a 0.1% protease inhibitor mixture (Cat. 11697498001, Roche, Germany). Protein concentrations were measured using a bicinchoninic acid protein assay kit (Cat. 23227, Thermo Fisher Scientific, United States). Proteins in the lysates were separated by sodium dodecyl sulfate-polyacrylamide gel electrophoresis and transferred to a polyvinylidene fluoride (PVDF) membrane (Millipore) prior to immunoblotting with anti-TMEM147 (ab97624, 1 : 1000, Abcam) and anti-*β*-actin (T40104M, 1 : 2000, Abmart) primary antibodies and the appropriate secondary antibodies.

### 2.9. Immunofluorescence Staining

Double immunofluorescence staining was performed using a multicolor fluorescence staining kit (10079100020, Panovue, China) according to the manufacturer's protocols. The primary antibodies used were rabbit anti-TMEM147 (ab97624, 1 : 200, Abcam), rabbit anti-NCAM1 (ab75813, 1 : 500, Abcam), and rabbit anti-F4/80 (70076S, 1 : 800, CST). Images were acquired with a fluorescence imaging system (Nikon, Eclipse Ts2, Japan).

### 2.10. Prognostic Model Construction and Prediction

HCC patients' clinical outcome data were downloaded from TCGA. Kaplan–Meier analysis was conducted to determine the clinical characteristics associated with overall survival (OS), disease specific survival (DSS), and progression-free interval (PFI). Survival curves were plotted in R (version 3.6.3) using the packages “survmine” and “survival”. Furthermore, Cox regression analysis was performed using the packages “survival” and “forestplot” in R [25]. The R package “rms” was used to construct nomograms and generate calibration plots [25]. In addition, receiver operating characteristic (ROC) analysis using the R packages “pROC” and “ggplot” was performed to assess the predictive accuracy of the model. The statistical analyses were performed with the significance level set at 0.05.

## 3. Results

### 3.1. TMEM147 Expression across Cancers and in HCC

**Figure 1:**
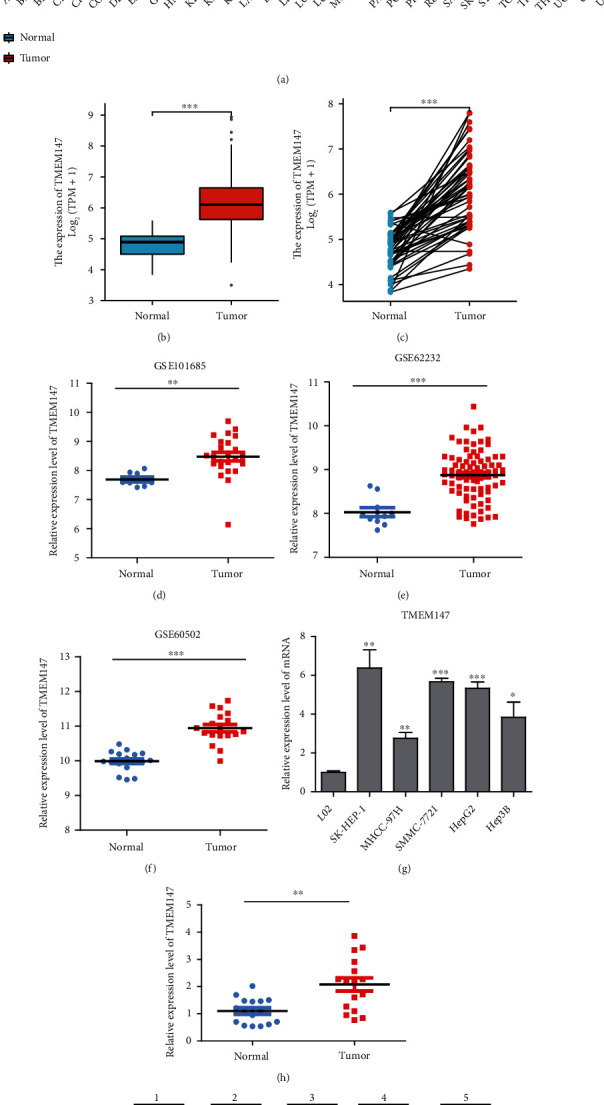
TMEM147 expression in various types of cancer. (a) The expression of TMEM147 in different tumor types. (b) TMEM147 expression levels in 374 HCC samples and 50 normal samples. (c) TMEM147 expression levels in HCC and paired normal tissues (*n* = 50). (d), (e), and (f) Expression of TMEM147 was verified in three GEO databases. (g) TMEM147 mRNA expression in five HCC cell line and normal liver epithelial cell line L02. (h) TMEM147 mRNA expression in HCC tissues and paired normal liver tissues (*n* = 16). (i) Western blotting detects TMEM147 protein level in HCC and paired normal tissues (*n* = 5) (∗*p* < 0.05, ∗∗*p* < 0.01, and ∗∗∗*p* < 0.001).

We used the Wilcoxon rank-sum test to compare the expression of TMEM147 among multiple cancer types according to TCGA data. TMEM147 expression was significantly higher in 25 types of cancer, including HCC, than in the corresponding normal tissues (Figure 1(a)). As shown in Figure 1(b), TMEM147 exhibited differential mRNA expression in tumor and normal tissues in the HCC dataset of TCGA. In paired samples, the expression of TMEM147 was substantially higher in the HCC samples than in the matched paracancerous samples (Figure 1(c)). We assessed TMEM147 expression in three different GEO datasets (GSE101685, GSE62232, and GSE60502) and validated the high expression level of TMEM147 in HCC (Figures 1(d), 1(e), and 1(f)). The expression of TMEM147 in HCC cell lines (SK-HEP-1, MHCC-97H, SMMC-7721, HepG2, and Hep3B) was significantly higher than in the normal liver epithelial cell line L02 (Figure 1(g)). In addition, we measured the mRNA and protein expression levels of TMEM147 in HCC and paired normal tissues, and the RT-qPCR and Western blot results confirmed the high expression of TMEM147 in HCC (Figures 1(h) and 1(i)).

### 3.2. Correlation between TMEM147 Expression and Clinicopathological Parameters

**Figure 2:**
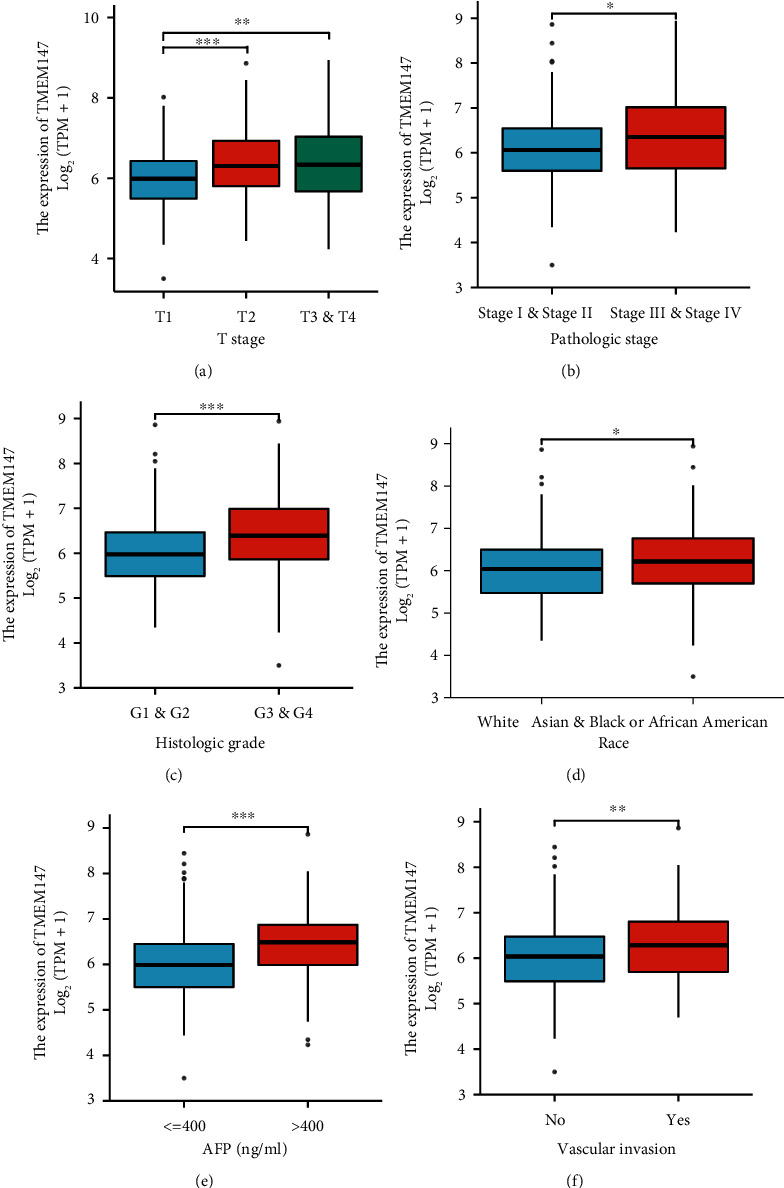
Box plots displaying TMEM147 expression in HCC patients according to various clinicopathological characteristics. (a) T stage; (b) pathologic stage; (c) histological grade; (d) race; (e) AFP, alpha-fetoprotein; and (f) vascular invasion (∗*p* < 0.05, ∗∗*p* < 0.01, and ∗∗∗*p* < 0.001).

To clarify the clinical significance of TMEM147 expression in HCC, we investigated the associations between TMEM147 expression and different clinical parameters. As shown in Table 1, overexpression of TMEM147 was correlated with higher T stage (T2 vs. T1, *p* < 0.001 and T3 & T4 vs. T1, *p* < 0.01), higher histological grade (*p* < 0.001), and more advanced pathological stage (*p* < 0.05) (Figures 2(a), 2(b), and 2(c)). In terms of race, TMEM147 was expressed at significantly higher levels in Whites than in Asians, Blacks, and African Americans (*p* < 0.05) (Figure 2(d)). HCC patients with alpha-fetoprotein (AFP) > 400 ng/mL had higher TMEM147 expression levels than those with AFP < 400 ng/mL (Figure 2(e)). Additionally, we found a positive association between TMEM147 expression and vascular invasion in HCC (Figure 2(f)). We further applied logistic regression analysis to confirm that TMEM147 expression was a dependent variable and correlated with poor prognostic factors. As shown in Table 2, the TMEM147 expression level in HCC was positively correlated with T stage (OR = 2.076, *p* < 0.001), pathological stage (OR = 1.862, *p* = 0.013), and histological grade (OR = 1.862, *p* < 0.001). These results indicated that TMEM147 expression affects HCC initiation and progression.

### 3.3. High TMEM147 Expression Is Correlated with Poor Prognosis in HCC Patients

**Figure 3:**
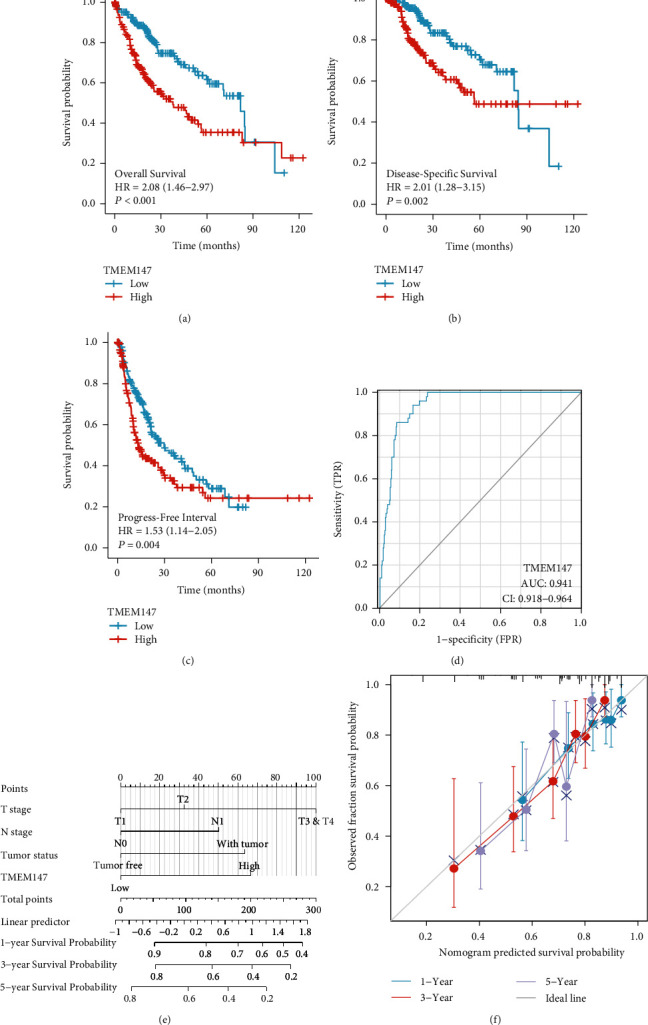
The efficacy of TMEM147 in the prognosis and prediction of HCC. (a) Analyses of OS with Kaplan–Meier; (b) DSS; (c) PFI; (d) the analysis of TMEM147 diagnostic efficacy for HCC using ROC curves; (e) nomogram to predict the 1-, 3-, and 5-year OS of the patient with HCC; (f) calibration plots validating the effectiveness of nomograms for OS.

We used Kaplan–Meier analysis to establish the relationship between TMEM147 expression and HCC patient prognosis. HCC patients with low TMEM147 expression had higher 10-year OS rates than those with higher TMEM147 expression (HR = 2.08, 95% CI = 1.46–2.97, *p* < 0.001) (Figure 3(a)). Prognostic analyses of DSS (HR = 2.01, 95% CI = 1.28–3.15, *p* = 0.002) (Figure 3(b)) and PFI (HR = 1.53, 95% CI = 1.14–2.05, *p* = 0.004) (Figure 3(c)) showed that patients in the high TMEM147 expression group had worse outcomes than those in the low TMEM147 expression group. ROC curves for HCC patients were generated to estimate the diagnostic value of TMEM147. Accordingly, the area under the curve (AUC) of TMEM147 was 0.941, indicating strong sensitivity and specificity for HCC diagnosis (Figure 3(d)). Moreover, the prognostic value of TMEM147 for HCC progression was further examined using multivariate Cox regression analysis. As Table 3 shows, TMEM147 overexpression was associated with worse OS in patients with HCC (HR: 2.121, *p* = 0.001), as well as with pathological stage (HR: 1.944, *p* = 0.017) and tumor status (HR: 1.977, *p* = 0.004). Univariate Cox regression analysis indicated that T stage (HR: 2.126, *p* < 0.001), M stage (HR: 4.077, *p* = 0.017), pathological stage (HR: 2.504, *p* < 0.001), tumor status (HR: 2.317, *p* < 0.001), and high expression of TMEM147 (HR: 2.079, *p* < 0.001) were independently correlated with poor outcomes in HCC patients.

### 3.4. Prognostic Model Generation in HCC

To better predict the prognosis of HCC patients, TMEM147 mRNA expression and other clinicopathological parameters were used to construct a prognostic nomogram. Four prognostic factors, namely, T stage, N stage, tumor status, and TMEM147 expression, were integrated into the TCGA data analysis. A point scale was applied using multivariate Cox analysis to score these variables. The total number of points for each variable was then calculated by rescaling the sum of each variable's points to a number between 0 and 100. A higher point total on the nomogram was positively correlated with a worse prognosis in HCC patients. A vertical line was drawn from the total score axis to the outcome axis to determine the 1-, 3-, and 5-year survival rates of patients with HCC. The survival probabilities of HCC patients at 1, 3, and 5 years were approximately 65%, 45%, and 25%, respectively (Figure 3(e)). The nomogram calibration curve for OS demonstrated good consistency in patients with HCC, which indicated that our prediction method was moderately accurate (Figure 3(f)).

### 3.5. Functional Enrichment Analysis of DEGs in HCC

**Figure 4:**
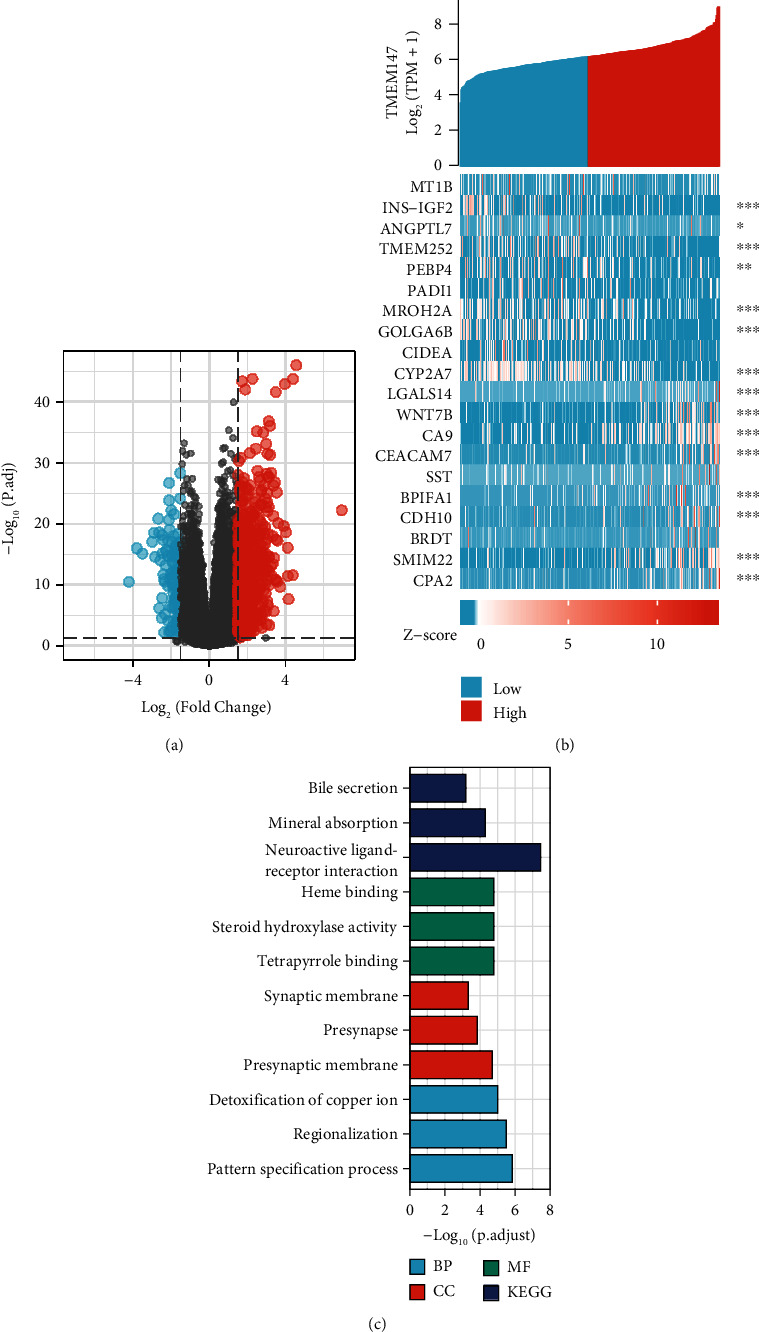
TMEM147 expression-related DEGs and GO/KEGG enrichment analysis for TMEM147. (a) Volcano plot of DEGs showing 743 genes up (red) and 133 down-regulated genes (blue); (b) heat map of the 20 DEGs, including 10 down-regulated genes and 10 upregulated genes; (c) top 3 enrichment related to DEGs with GO/KEGG enrichment analysis (∗*p* < 0.05, ∗∗*p* < 0.01, and ∗∗∗*p* < 0.001).

**Figure 5:**
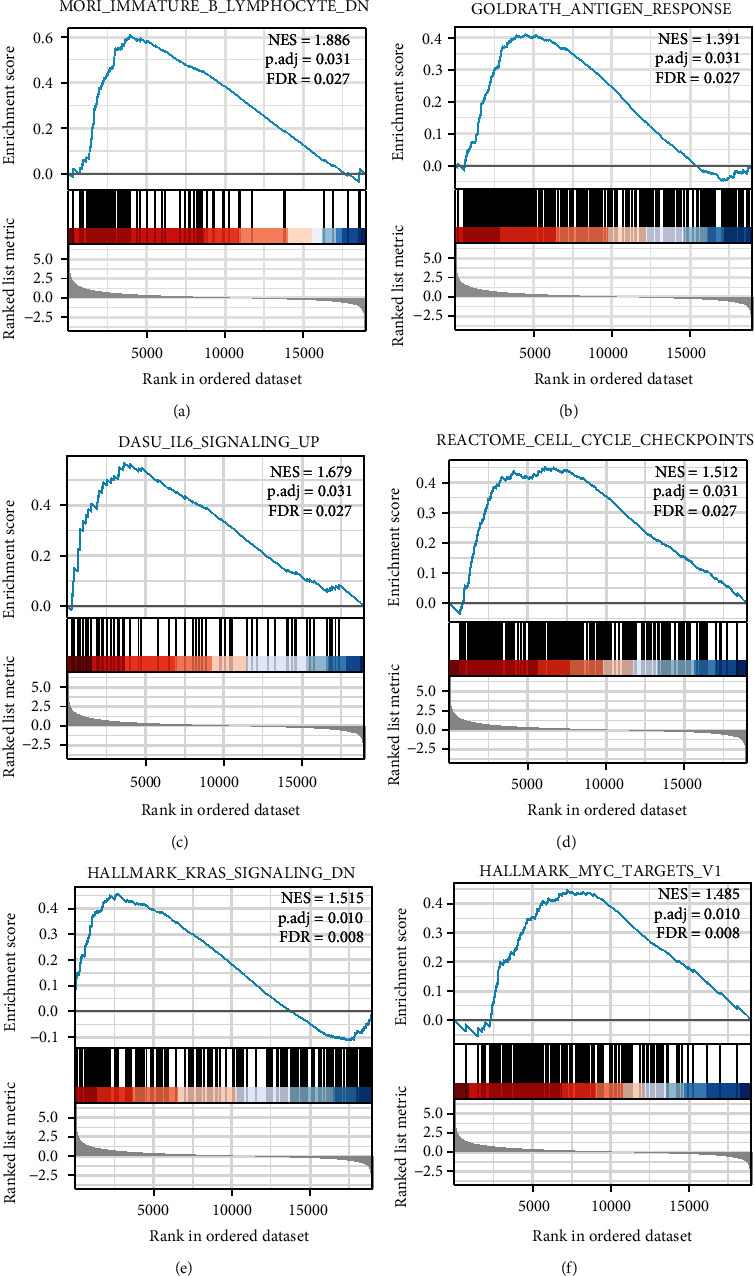
Enrichment plots from GSEA. TMEM147 was differentially enriched in (a) B lymphocyte, (b) antigen response, (c) IL6 signaling pathway, (d) cell cycle, (e) KRAS signaling pathway, and (f) MYC targets.

To explore the mechanisms by which abnormally high TMEM147 expression promotes HCC progression, we compared gene expression in HCC tissues from patients with high and low TMEM147 expression. There were 743 DEGs, among which 610 were downregulated and 133 were upregulated (adjusted *p* < 0.05, |Log2-fold change| > 1.5) (Figure 4(a)). Heatmaps were generated to show the downregulated and upregulated DEGs in order of prominence (Figure 4(b)). GO term and KEGG pathway enrichment analyses were used to further predict the functions of the TMEM147-related genes in HCC patients (Figure 4(c)). According to GO term enrichment analysis, the TMEM147-related genes were enriched in various BP, cell component (CC), and MF terms, including pattern specification process, regionalization, detoxification of copper ion, presynaptic membrane, presynapse, synaptic membrane, tetrapyrrole binding, steroid hydroxylase activity, and heme binding. Furthermore, KEGG pathway enrichment analysis showed TMEM147-related genes to be enriched in the neuroactive ligand–receptor interaction, mineral absorption, and bile secretion pathways. To identify TMEM147-related signaling pathways in HCC, we performed GSEA of the low and high TMEM147 expression datasets from the Molecular Signatures Database (MSigDB) (C2.all.http://v7.2.symbols.gmt and h.all. v7.2.symbols.gm, adjusted *p* < 0.05, FDR < 0.25). The gene sets enriched in the high TMEM147 expression group included B lymphocyte, antigen response, IL6 signaling pathway, cell cycle, Kirsten rat sarcoma viral oncogene homolog (KRAS) signaling pathway, and myelocytomatosis oncogene (MYC) targets (Figures 5(a), 5(b), 5(c), 5(d), 5(e), and 5(f)).

### 3.6. Analysis of the Correlations between TMEM147 Expression and Immune Cell Infiltration in HCC

**Figure 6:**
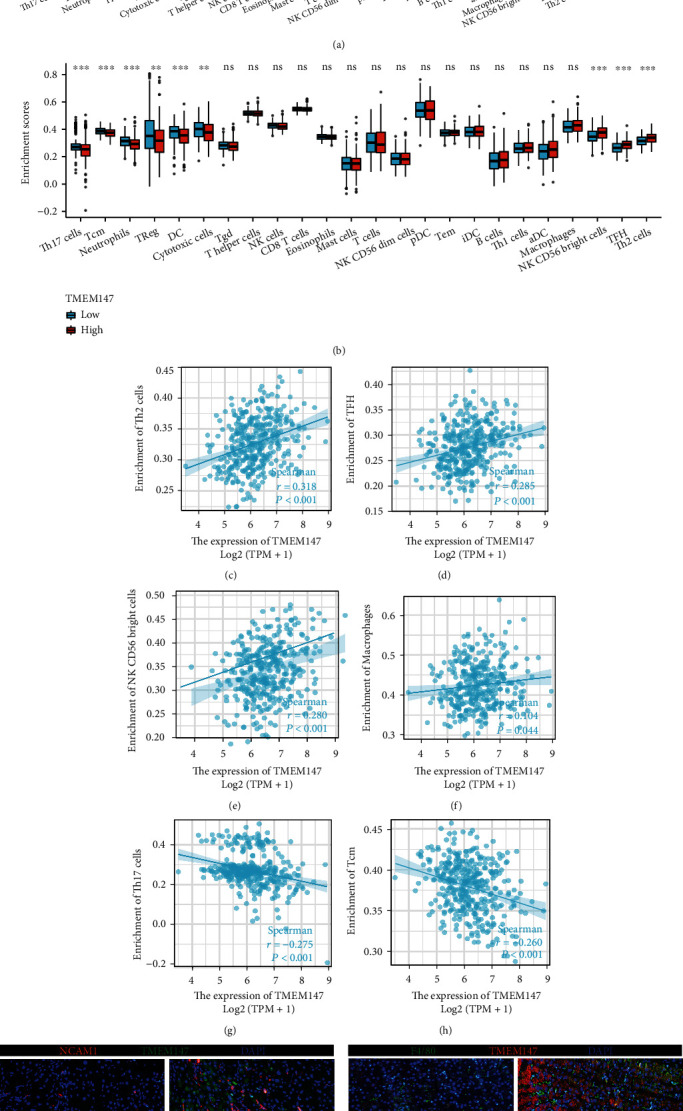
Association between the expression of TMEM147 and immune cell infiltration in the HCC microenvironment. (a) and (b) Relationship between TMEM147 expression and infiltration of 24 immune cell types; TMEM147 expression strongly positively associates with infiltrating levels of (c) Th2 cells, (d) TFH cells, (e) NK CD56 bright cells, and (f) macrophages; TMEM147 has a negative correlation with (g) Th17 cells and (h) Tcm cells; immunofluorescence co-staining for NCAM1 and TMEM147 (i), as well as F4/80 and TMEM147 (j) in HCC and paired normal tissues (scale bar, 20 *μ*m).

Considering that GSEA showed that there may be an association between TMEM147 and tumor immunity, ssGSEA was used to test whether TMEM147 expression correlates with immune infiltration in HCC (Figures 6(a) and 6(b)). According to the results, TMEM147 overexpression was significantly positively correlated with infiltration of Th2 cells (*R* = 0.318, *p* < 0.001), follicular helper T (TFH) cells (*R* = 0.285, *p* < 0.001), NK CD56 bright cells (*R* = 0.280, *p* < 0.001), and macrophages (*R* = 0.104, *p* < 0.044) (Figures 6(c), 6(d), 6(e), and 6(f)). In contrast, TMEM147 expression had a strong negative correlation with Th17 cells (*R* = −0.275, *p* < 0.001) and central memory T (Tcm) cells (*R* = −0.260, *p* < 0.001) in HCC (Figures 6(g) and 6(h)). Consistent with the bioinformatics analysis results, the immunofluorescence staining results demonstrated marked expression of NCAM1 (a marker of NK CD56 bright cells) and F4/80 (a marker of macrophages) in human HCC tissues with high TMEM147 expression (Figures 6(i) and 6(j)).

## 4. Discussion

HCC is one of the most common and deadly cancers worldwide [26]. Despite advances in early diagnosis, new targeted drugs, and immune checkpoint inhibitors, HCC patients have a 5-year survival rate of only 18.1%, as most patients have late-stage disease at initial diagnosis [27]. Thus, it is crucial to identify potential prognostic biomarkers of HCC to improve patient outcomes. In our study, TMEM147 mRNA expression was found to be upregulated in HCC tissues. Additionally, HCC patients with high TMEM147 expression had worse prognoses and shorter survival times. Preliminary mechanistic studies revealed that TMEM147 promotes HCC progression in a manner possibly associated with immune infiltration. Our study implies that TMEM147 may be a promising target for HCC treatment.

TMEM147 is a seven-transmembrane protein that significantly impacts the pathology of different diseases and aberrant processes, including tumorigenesis [9, 12]. Research has revealed that colon cancer exhibits high mRNA expression of TMEM147, which could thus be involved in the pathogenesis of colon cancer [13]. However, TMEM147 expression and its prognostic value in HCC are unclear. In the present study, through analysis of public TCGA data, we found that TMEM147 was highly expressed in HCC. To verify the above results, we investigated the expression of TMEM147 in HCC samples and HCC cell lines by RT-qPCR and Western blotting. We found that TMEM147 mRNA expression was significantly increased in HCC tissues and HCC cell lines. This finding indicates that TMEM147 could be considered a diagnostic biomarker for HCC. Furthermore, ROC curve analysis revealed that TMEM147 expression was a reliable diagnostic marker for differentiating HCC tissues from normal liver tissues, with an AUC of 0.941. In addition, higher expression of TMEM147 was significantly correlated with several clinicopathological features, including higher T stage, pathological stage, and histologic grade; higher AFP; White race; and vascular invasion. Based on our findings, we concluded that the expression of TMEM147 could be a novel diagnostic biomarker for HCC.

HCC patients with high TMEM147 expression have poor prognoses. By Kaplan–Meier analysis, we investigated whether high TMEM147 expression is associated with significantly worse OS, worse DSS, and a shorter PFI in HCC. Multivariate Cox analysis showed that pathological stage (Stage III & IV vs. Stage I & II), tumor status (with tumor vs. tumor free), and TMEM147 expression (high) were independent prognostic factors for OS. In parallel, we constructed a prognostic nomogram based on T and N classification, tumor status, and TMEM147 expression in HCC. According to the calibration plot, the predicted and actual probabilities for 1-, 3-, and 5-year OS agreed well. Thus, the prognostic nomogram based on TMEM147 expression might be clinically valuable for patients with HCC.

Given the close association between TMEM147 expression and HCC progression, we sought to explore the possible functions and mechanisms of TMEM147 in HCC. GSEA indicated that the TMEM147-high phenotype was associated with the MYC targets and KRAS signaling pathway gene sets. Recent studies have revealed that the activation of MYC can contribute to HCC progression by promoting cell proliferation, metastasis, and angiogenesis [28]. There is also evidence that KRAS pathway activation contributes to the initiation and progression of HCC [29]. Therefore, TMEM147 expression might be necessary for HCC tumorigenesis. Interestingly, GSEA also showed that TMEM147 may be involved in signaling pathways that regulate immune and inflammation in HCC, including the B lymphocyte, antigen response, and IL6 signaling pathway gene sets. Our bioinformatics analysis and immunofluorescence staining indicated that high TMEM147 expression was positively associated with infiltration of Th2 cells, TFH cells, NK CD56 bright cells, and macrophages. Th2 cell infiltration has been associated with immunosuppression and poor survival in a number of malignancies [30, 31]. In a mouse model of HCC, TFH cell infiltration showed a similar negative correlation to Th2 cell infiltration with regard to survival [32]. Studies on breast, colorectal, and lung cancers have shown that NK CD56 bright cells have very high infiltration levels and promote tumorigenesis [33–35]. Macrophages are among the most abundant immune cells infiltrating the tumor microenvironment (TME) in liver cancer and are present at all stages of liver cancer progression [36]. According to earlier research, osteosarcomas with higher TMEM147 expression have less M2 macrophage infiltration and a worse prognosis [14]. In addition to macrophages, neutrophils, DCs, and T cells had significantly different abundances in osteosarcomas with high and low TMEM147 expression, indicating that TMEM147 may mediate the immune microenvironment in osteosarcomas [14]. In fact, the TMEM protein family is heavily involved in the development and immune infiltration of many tumors. For instance, high expression of TMEM204 is linked to high levels of CD8+ and CD4+ T cell, macrophage, neutrophil, and bone marrow dendritic cell infiltration in liver hepatocellular carcinoma (LIHC) [37]. Based on these results, we speculated that TMEM147 may play an essential role in the progression of HCC through a mechanism that regulates immune-related pathways to promote immune infiltration.

Although our work indicates the high expression of TMEM147 and its relationship with HCC progression, several key predictions require further functional validation. For instance, our clinical sample size is constrained, and bigger clinical investigations are required to confirm if TMEM147 expression is a prognostic and predictive factor for HCC. In addition, future work is required to design *in vivo/in vitro* studies to investigate how TMEM147 affects immune infiltration in HCC.

## 5. Conclusion

In summary, we found that high TMEM147 expression is closely correlated with poor prognosis in patients with HCC in this study. Overexpression of TMEM147 could play a vital role in the progression of HCC by increasing the infiltration of Th2 cells, TFH cells, NK CD56 bright cells, and macrophages. These findings support TMEM147's role as a prognostic biomarker for HCC via mediation of immune infiltration.

## Figures and Tables

**Table 1:** Association of TMEM147 expression and clinicopathological features in HCC (*n* = 374).

Characteristic	Level	Low expression of TMEM147	High expression of TMEM147	*p*	Method
*N*		187	187		
T stage, *n* (%)	T1	108 (29.1%)	75 (20.2%)	0.005	Chisq. test
	T2	41 (11.1%)	54 (14.6%)		
	T3	30 (8.1%)	50 (13.5%)		
	T4	6 (1.6%)	7 (1.9%)		
N stage, *n* (%)	N0	126 (48.8%)	128 (49.6%)	0.122	Fisher test
	N1	0 (0%)	4 (1.6%)		
M stage, *n* (%)	M0	128 (47.1%)	140 (51.5%)	0.624	Fisher test
	M1	1 (0.4%)	3 (1.1%)		
Pathologic stage, *n* (%)	Stage I	103 (29.4%)	70 (20%)	0.004	Fisher test
	Stage II	38 (10.9%)	49 (14%)		
	Stage III	34 (9.7%)	51 (14.6%)		
Tumor status, *n* (%)	Tumor free	110 (31%)	92 (25.9%)	0.163	Chisq. test
	With tumor	71 (20%)	82 (23.1%)		
	Stage IV	1 (0.3%)	4 (1.1%)		
Gender, *n* (%)	Female	54 (14.4%)	67 (17.9%)	0.185	Chisq. test
	Male	133 (35.6%)	120 (32.1%)		
Race, *n* (%)	Asian	72 (19.9%)	88 (24.3%)	0.278	Chisq. test
	Black or African American	9 (2.5%)	8 (2.2%)		
	White	99 (27.3%)	86 (23.8%)		
Age, *n* (%)	≤60	83 (22.3%)	94 (25.2%)	0.323	Chisq. test
	>60	103 (27.6%)	93 (24.9%)		
Weight, *n* (%)	≤70	78 (22.5%)	106 (30.6%)	0.002	Chisq. test
	>70	96 (27.7%)	66 (19.1%)		
BMI, *n* (%)	≤25	77 (22.8%)	100 (29.7%)	0.019	Chisq. test
	>25	91 (27%)	69 (20.5%)		
Histologic grade, *n* (%)	G1	35 (9.5%)	20 (5.4%)	<0.001	Chisq. test
	G2	102 (27.6%)	76 (20.6%)		
	G3	43 (11.7%)	81 (22%)		
	G4	5 (1.4%)	7 (1.9%)		
AFP (ng/ml), *n* (%)	≤400	123 (43.9%)	92 (32.9%)	<0.001	Chisq. test
	>400	20 (7.1%)	45 (16.1%)		
Vascular invasion, *n* (%)	No	118 (37.1%)	90 (28.3%)	0.007	Chisq. test
	Yes	44 (13.8%)	66 (20.8%)		
Fibrosis Ishak score, *n* (%)	0	47 (21.9%)	28 (13%)	0.343	Chisq. test
	1/2	17 (7.9%)	14 (6.5%)		
	3/4	12 (5.6%)	16 (7.4%)		
	5/6	45 (20.9%)	36 (16.7%)		
Adjacent hepatic tissue inflammation, *n* (%)	None	76 (32.1%)	42 (17.7%)	0.027	Chisq. test
	Mild	47 (19.8%)	54 (22.8%)		
	Severe	11 (4.6%)	7 (3%)		
OS event, *n* (%)	Alive	137 (36.6%)	107 (28.6%)	0.002	Chisq. test
	Dead	50 (13.4%)	80 (21.4%)		
Age, median (IQR)		62 (54, 69)	60 (50, 68)	0.123	Wilcoxon

BMI: body mass index; AFP: alpha-fetoprotein; OS: overall survival; IQR: inter-quartile range.

**Table 2:** Logistic analysis of the relationship between clinicopathologic features of HCC and the expression of TMEM147.

Characteristics	Total (*N*)	Odds ratio (OR)	*P* value
T stage (T2 & T3 & T4 vs. T1)	371	2.076 (1.375–3.149)	<0.001
N stage (N1 vs. N0)	258	72589629.123 (0.000–NA)	0.994
M stage (M1 vs. M0)	272	2.743 (0.346–55.834)	0.385
Pathologic stage (Stage III & Stage IV vs. Stage I & Stage II)	350	1.862 (1.147–3.056)	0.013
Histologic grade (G3 & G4 vs. G1 & G2)	369	2.616 (1.695–4.075)	<0.001
Tumor status (with tumor vs. tumor free)	355	1.381 (0.907–2.108)	0.133
Fibrosis Ishak score (3/4 & 5/6 vs. 0 & 1/2)	215	1.390 (0.810–2.395)	0.233
Residual tumor (R1 & R2 vs. R0)	345	1.640 (0.630–4.555)	0.318

**Table 3:** A multivariate and univariate Cox proportional hazards analysis of TMEM147 expression in HCC.

Characteristics	Total (*N*)	Univariate analysis		Multivariate analysis	
		Hazard ratio (95% CI)	*P* value	Hazard ratio (95% CI)	*P* value
T stage (T2 & T3 & T4 vs. T1)	370	2.126 (1.481–3.052)	<0.001	1.433 (0.798–2.574)	0.228
N stage (N0 vs. N1)	258	2.029 (0.497–8.281)	0.324		
M stage (M1 vs. M0)	272	4.077 (1.281–12.973)	0.017	1.208 (0.287–5.079)	0.797
Pathologic stage (Stage I & Stage II vs. Stage III & Stage IV)	349	2.504 (1.727–3.631)	<0.001	1.944 (1.128–3.351)	0.017
Tumor status (tumor free vs. with tumor)	354	2.317 (1.590–3.376)	<0.001	1.977 (1.239–3.155)	0.004
Gender (female vs. male)	373	0.793 (0.557–1.130)	0.2		
Race (White vs. Black or African American)	361	0.791 (0.551–1.135)	0.203		
Age (≤0 vs. >60)	373	1.205 (0.850–1.708)	0.295		
Weight (≤70 vs. >70)	345	0.941 (0.657–1.346)	0.738		
BMI (≤25 vs. >25)	336	0.798 (0.550–1.158)	0.235		
Histologic grade (G1 & G2 vs.G3 & G4)	368	1.091 (0.761–1.564)	0.636		
AFP (ng/ml) (≤400 vs. >400)	279	1.075 (0.658–1.759)	0.772		
Vascular invasion (no vs. yes)	317	1.344 (0.887–2.035)	0.163		
Fibrosis Ishak score (0 & 1/2 vs. 3/4 & 5/6)	214	0.740 (0.445–1.232)	0.247		
Adjacent hepatic tissue inflammation (none vs. mild & severe)	236	1.194 (0.734–1.942)	0.475		
TMEM147 (low vs. high)	373	2.079 (1.458–2.966)	<0.001	2.121 (1.336–3.369)	0.001

TMEM147: transmembrane protein 14.

## Data Availability

The data underlying this study are freely available from the TCGA dataset (https://portal.gdc.cancer.gov/). The data sets used and/or analyzed during the current study are available from the corresponding author on reasonable request.
